# Exploring gender perceptions of risk of HIV infection and related behaviour among elderly men and women of Ga-Rankuwa, Gauteng Province, South Africa

**DOI:** 10.1080/17290376.2016.1218790

**Published:** 2016-08-15

**Authors:** Eucebious Lekalakala-Mokgele

**Affiliations:** ^a^ PhD, is Director of the School of Health Care Sciences, Sefako Makgatho Health Sciences University, Pretoria, South Africa

**Keywords:** HIV/AIDS, gender, perceptions, elderly, Perceptions des, personnes âgées, sur le genre, VIH/SIDA

## Abstract

The literature shows that there are important differences between women and men in the underlying mechanisms of transmission of HIV infection and AIDS, as well as in the social and economic consequences of HIV/AIDS. These stem from sexual behaviour and socially constructed ‘gender’ differences between women and men in roles and responsibilities. Despite the fact that numerous gender-related sociocultural factors influence HIV/AIDS protective behaviours, little gender specificity is included in HIV prevention among the elderly. In order to close this gap, this study explored gender-related perceptions of risk of HIV infection among elderly men and women of Ga-Rankuwa in Gauteng Province, South Africa. This qualitative study used purposive sampling to conduct three focus group interviews with 22 women and 10 men who were above 60 years of age. Findings revealed that both genders blame each other for the spreading of HIV/AIDS. Male participants displayed the tendency to have multiple partners, whereas females accepted that males are promiscuous. Mixed perceptions about disclosure of HIV status were found. Condom use was a challenge, as men did not know how to introduce it with their wives, and some female participants indicated that men are resistant to using condoms. The elderly men also believed that women will have sex in exchange for money. It is concluded that there is a need for substantial behaviour change among both elderly males and females, which should address gender power relations. More in-depth and extensive research in this area is recommended.

## Résumé

La littérature montre qu'il existe des différences importantes entre les femmes et les hommes dans les mécanismes sous-jacents la transmission de l'infection au VIH et le SIDA, ainsi que dans les conséquences sociales et économiques du VIH / SIDA. Ceux-ci proviennent des comportements sexuels et des différences socialement construits au tour du «genre» entre les femmes et les hommes dans leur rôles et responsabilités. Malgré le fait que de nombreux facteurs socioculturels liés au genre ont de l’influence sur les comportements de protection contre le VIH / SIDA, peu de facteurs liés au spécificité du genre sont inclus dans la prévention du VIH chez les personnes âgées. Afin de combler cette lacune, la présente étude a exploré les perceptions de risque à l’infection du VIH/SIDA relatives au genre chez les hommes et les femmes âgées de Ga-Rankuwa dans la province de Gauteng, Afrique du Sud. Cette étude qualitative a utilisé un échantillonnage raisonné pour mener des entrevues avec trois groupes focalisés de 22 femmes et 10 hommes qui étaient au-dessus de 60 ans. Les résultats ont révélé que les deux sexes se blâment mutuellement pour la propagation du VIH / SIDA. Les participants masculins affichent la tendance à avoir des partenaires multiples, alors que les femmes avouent la promiscuité des hommes. Les perceptions confondues sur la divulgation du statut du VIH ont été trouvées. L'utilisation du préservatif était un défi, car les hommes ne savent pas comment l'utuliser avec leurs épouses, et certains participants féminins ont indiqué que les hommes sont réticents à utiliser des préservatifs. Les hommes âgés croyaient aussi que les femmes vont avoir des relations sexuelles en échange d'argent. Il est conclu qu'il existe un besoin important de changement de comportement qui pourrait répondre au problème du pouvoir de relation lié au genre. Une recherche plus approfondie et de grande etandue est recommendée dans ce domaine.

## 1. Introduction

Gender roles and relations are increasingly recognised as one of the fundamental forces driving the rapid spread of HIV infection and exacerbating the impact of AIDS. According to Anderson, Beutel and Maughan-Brown (2007), gender-based beliefs, pressures, roles and power may influence the ability of males and females to behave in ways that correspond to their risk perception. The stereotypical behaviour of some men, together with unfavourable cultural prescriptions such as submissiveness, sexual subordination, obedience and willingness, make women in particular more vulnerable to becoming infected with HIV (Van den Berg & Van Rooyen 2007).

Behavioural, psychological and sociocultural factors make individuals more or less vulnerable to HIV infection (FaIola & Heaton 2007; Scott 2009), and the elderly are no exception. Evidence on the social construction of manhood suggests that the health of males is indirectly affected by risky behaviour, increasing their female partners’ vulnerability to sexually transmitted diseases, including HIV (Peacock, Redpath, Weston, Evans, Daub & Greig 2008). According to Medjuck (2008), although women face biological vulnerability to HIV infection, many of the underlying factors relating to the high rates of HIV infection among them are socially constructed. Many authors are of the opinion that patriarchal African culture as well as gender inequalities play a significant role in the transmission of HIV/AIDS (Deribe, Woldemichael, Njau, Yakob, Biadgilign & Amberbir 2010; Mofolo 2010).

In some African societies, male domination and superiority along with female submission and inferiority are perpetuated by patriarchy, and women are looked upon as perpetually dependent beings who always have to be directed and protected by men (Baloyi 2010). It is within the confines of the same African cultures that the view that women should be regarded as objects of sexual fulfilment is endorsed, and men are encouraged to see women in this light (Baloyi 2009).

Dominant ideologies of masculinity encourage men to demonstrate sexual prowess by having multiple partners and sexual entitlement to women, thus contributing to men’s risky sexual practices (Stern, Rau & Cooper 2014). Multiple partnerships are condoned and even encouraged for men, while women are expected to be monogamous and unquestioning of their partner’s behaviour and this facilitates the spreading of the virus in South Africa (Mswela 2009:175). According to Van Staden and Badenhorst (2009), just as male dominance influences the sexual behaviour of females and places them at risk, so do cultural practices such as the male view of perceived masculinity also increases the male risk of contracting HIV infection. The same gender roles and relations that enhance women’s vulnerabilities to HIV/AIDS also increase some of the risks for men.

Across the sub-Saharan African region, gender-related norms all too often grant men the power to initiate and dictate the terms of sex, making it extremely difficult for women to protect themselves from either HIV or any other sexually transmitted diseases (Farrar 2013; Peacock *et al*. 2008; Versteeg & Murray 2008). While condom use has become more common in South Africa in response to the AIDS epidemic, gender norms continue to limit their use among some men who see health-seeking behaviour as weak, and among women, who may be seen as ‘easy’ and unfaithful if they carry condoms (Peacock *et al*. 2008)

In many countries, women and girls are bearing a heavier burden than men in terms of the rate of HIV infection and the stigmatisation that results from their being blamed for spreading HIV/AIDS (Petros, Airhihenbuwa, Simbayi, Ramlagan & Brown 2006; Rankin, Brennan, Schell, Laviwa & Rankin 2005). Intergenerational sexual relations are thought to play an important role in the propagation of HIV infection in Africa. HIV prevalence disparities by gender and age may be thought of as a complex interplay between many factors, including multiple and concurrent partnerships, age differences between partners, and power imbalances (Wyrod *et al*. 2011).

A study in Botswana shows that elderly men are more likely to have sexual partners who are ten years younger than they are and are at risk of being infected with the virus (Keetile 2014). The same men then infect their elderly female partners after having unprotected sex with younger females (Smith 2002). Older women are further at higher risk of contracting HIV because of menopausal changes in the vaginal mucosa, increasing the likelihood of trauma and sexually transmitted diseases (Nguyen & Holodniy 2008; Ramjee & Daniels 2013). Although physiology heightens women’s risk of transmission, it is women’s relative lack of power over their bodies and sexual lives, supported and reinforced by their social and economic inequality, that renders them vulnerable to contracting HIV and having to cope with HIV/AIDS (Gupta & Weiss 1996).

South African men are also much less likely than women to present for HIV testing, and therefore less likely to be aware of their status. This reluctance imperils their female partners, who may be less likely to use HIV prevention methods if unaware that their partners are HIV-positive (Peacock *et al*. 2008). The power imbalance between men and women also translates into economic dependence of women (Kenyon, Boulle, Badri & Asselman 2010). Women’s limited economic options and relative powerlessness may force them into sex work in order to cope with household economic crises (Smith 2002).

Research on gender perceptions of risk of HIV infection has been carried out among younger generations, but has not been explored in the elderly population. The purpose of this study was therefore to explore gender perceptions of risk of HIV infection in this population.

## 2. Methods

### 2.1. Study setting

The study was conducted in Ga-Rankuwa, a township located about 37 km north of Pretoria in Gauteng Province. It is a diverse township whose residents speak many languages. Ga-Rankuwa was chosen as the setting because of the researcher’s interest in gerontology and membership of community forum on geriatric care in this setting. Focus group interviews were conducted in luncheon clubs for the elderly in the community. A luncheon club is a place – usually a church building, clubhouse, recreation hall or a home – where elderly people aged 50 and above meet once or twice a week, the purpose being to create social companionship and help to meet the emotional, cultural, physical, spiritual and educational needs of the aged. These clubs are run by old people themselves in partnership with community groups and a social worker.

### 2.2. Research strategy

The study was located within a qualitative, exploratory, descriptive research paradigm because of its appropriateness for unravelling subjective phenomena such as the gender perceptions of older persons. Qualitative research explores phenomena under scrutiny by focusing on the participants, and it is committed to the participants’ viewpoint (Streubert & Carpenter 2011:20). This research therefore does not attempt to make a generalisable conclusion about gender perceptions, but rather is aimed at collecting adequate data from a variety of people to enable identification of key emergent themes, insights and understandings of the phenomenon of gender perceptions of the risk of HIV infection among older persons.

### 2.3. Recruitment

The researcher is active in the community activities of the elderly, and visited luncheon clubs to recruit participants. A slot was given to the researcher at the end of the monthly meeting at each club to introduce herself and explain the purpose of the research. After explaining the study to eligible potential participants, the chairperson of each luncheon club notified those who were willing to participate that they would be contacted by the researcher to set up a suitable time and venue for conducting a focus group discussion (FGD).

### 2.4. Sampling

The study utilised purposive non-probability criterion sampling, which involves searching for individuals who meet a certain criterion (Palys 2008). In this study, the participants had to be aged 60 years and above. A sample of 32 individuals was deemed large by qualitative standards and sufficient to explore a wide range of perceptions. Qualitative inquiry works with small samples of participants who are selected purposefully to permit understanding of a particular phenomenon in depth. The thrust of qualitative studies is neither the numerical distribution of study participants among factors nor the number of people that hold a particular view, but on how and why people hold the beliefs that they do (Judgeo & Moalusi 2014).

### 2.5. Participants

FGDs were conducted with 22 elderly women and 10 men. It was important to have both genders represented in order to be able to explore this phenomenon. The larger representation of women was due to the fact few men make use of luncheon clubs. Table 1 presents the age, sex, language and educational level of participants. All interviews were conducted in luncheon clubs after the elderly completed their activities.

**Table 1.** Demographic data of the 32 older persons interviewed.

	Number	%
*Age (years)*		
60–70	12	37.5
71–80	11	34.4
81–90	9	28.1
*Ethnic language*		
Setswana	25	78.1
Sesotho	1	3.1
Sepedi	2	6.3
Sizulu	4	12.5
*Gender*		
Females	22	68.8
Males	10	31.2
*Marital status*		
Single	3	9.4
Married	20	62.5
Widow	7	21.8
Cohabiting	2	6.3
*Level of education*		
No formal education	11	34.3
Primary education	11	34.3
Secondary education	4	12.5
High school	2	6.3
Tertiary	4	12.5

### 2.6. Data collection

Three gender-segregated FGDs were conducted as follows: a group of 12 females, another of 10 females, and a group of 10 male participants. Interviews were conducted in languages as preferred by all participants. Some participants responded in English as they chose to do so. A skilled qualitative data facilitator collected data in the language of choice of participants, while the researcher wrote field notes to record non-verbal cues and details. In addition to biographical questions which were completed by the researcher, an FGD guide was designed to enable the facilitator to probe similar issues with participants. The main question asked in each group was: What do you think put you at risk of being infected with HIV? The guide comprised key questions and probes designed to encourage participants to express their perceptions regarding the risks of HIV infection and the roles of each gender. All interviews were audio-recorded, transcribed and translated into English.

### 2.7. Data analysis

The data were analysed using a thematic approach, which aims to understand an issue by revealing the prominent themes at various levels in a text in order to provide a holistic account of the data (Vaismoradi, Jones, Turunen & Snelgrove 2016). Multiple readings of the transcripts were carried out by the researcher to familiarise herself with content and meaning. Data were then analysed manually to identify emerging themes and subthemes that reflected gender perceptions of elderly female and male participants. This was followed by developing a code list. Quotes relevant to each theme were extracted from the data. These reflected divergent perceptions of risk of HIV infection from both female and male elderly participants.

### 2.8. Enhancing rigour

Research questions were informed by a comprehensive literature review. The sample size was adequate to allow for emergence of diverse viewpoints. Addressing participants in a large group during recruitment assisted in diminishing selection bias, as only those willing to participate offered to do so, without any coercion. Participants were allowed to use their preferred ethnic language in responding to research questions. The skilled facilitator was also multilingual. Audio-recordings of interviews ensured that data were correctly and precisely captured. Both the researcher and facilitator analysed the data, including the coding process, which allowed for interrogation of interpretation.

### 2.9. Bias

Sampling bias was avoided by carefully selecting participants who met the age criterion and represented the group of interest. Biased questioning was avoided by redirecting questions to other participants during probing, and use of an independent coder assisted in prevention of interpretation bias.

### 2.10. Ethical considerations

Permission to conduct the study was obtained from Sefako Makgatho University Research and Ethics Committee (Reference number: MREC/H/305/2013). Approval for the study was granted by the managers of the luncheon clubs and the elderly persons before data collection. The nature and scope of the study were explained to the participants, who gave their informed consent. They were informed that participation was voluntary and that they had the right to discontinue their participation if they felt uncomfortable with the topic under discussion or did not wish to continue. All participants were assured that confidentiality would be maintained. This was assured as only the researcher had access to the audiotaped material, which was kept locked up in a safe place and would be erased after the research was completed. Use of real names during data analysis was avoided.

## 3. Findings

Five themes emerged from the interviews: blaming, lack of disclosure, condom use, male **p**rowess and double standards, and economic status.

### 3.1. Blaming

The findings show that both female and male participants blamed each other for the spread of HIV infection. A male participant feels that females are responsible for spreading HIV infection. The women are of the opinion that men are unfaithful and are responsible for infecting women:

I want to tell you something now, which is a fact … there are people who are cruel, especially ladies, I apologise ladies … going around in trains, in shebeens, where there is fun. This person goes around carrying the disease, spreading it. (Male, 78 years)

We men, as we were talking earlier about elderly people, that they also get infected. Some men go to shebeens where these women are and pay R20, R40, to get a quick one. There are those women, some use the train, and they spread the disease. I do not know how government can get a hold of the people … if they can arrest them, this disease will end. (Male, 70 years)

It’s older men who run around with young girls … (Female, 65 years)

These days, we cannot trust each other as people … you can trust your partner, whereas they are being unfaithful, and then infect you. (Female, 67 years)

Let’s say in my situation … I stay with my old man, I take it that we’re both old, and I have dedicated myself to him, only to find that he is fooling around out there. When he is with me, he doesn’t even use condoms, thereby exposing you to HIV, while you are faithful. (Female, 68 years)

### 3.2. Disclosure

Perceptions on HIV disclosure varied by gender. Male participants had divergent views among themselves; some cited denial, while others were open to disclosure:

An infected person will never disclose it, especially when they haven’t tested … they just go on, they won’t disclose. (Male, 74 years)

I think we don’t want to accept it and declare openly, we think it will disappear. When people disclose, as man you should tell your partner what you have been up to, but now people don’t disclose … that’s it. (Male, 71 years)

When you have it, you must talk about it, so you won’t infect other people. You have to declare your status these days. Previously those things would bring shame, but today you have to speak out, so people know that you have the virus. (Male, 81 years)

One of the male participants indicated that it was even difficult to disclose to their male friends, as the latter would gossip about them:

Men these days gossip so much … to a point where ladies would be waiting for them to come with the news, so that is why they hide the disease … (Male, 68 years)

Female participants were open to disclosure – provided that it is done by their doctors:

I’d ask him to accompany me inside, then ask the doctor to please conduct blood tests on the both of us. He won’t refuse. Then when the results come back, we both get to find out our statuses. (Female, 70 years)

The doctor will have to test us both, because we’re married. If I’m sick as his wife, then he also has to get tested. We will be there to receive our results together. If I’m the infected one, the results will show. If he is infected, it will also show. (Female, 68 years)

### 3.3. Condom use

Participants indicated that it is difficult to introduce condoms at their age, as they have never done so earlier with their partners. Men were concerned about exposure of their unfaithfulness, and said that they would question their wives if they were to introduce a condom:

As old as I am, how can I go and introduce a condom at home? My wife will be surprised, and wonder what I’m trying to do now. Even if I can get them for free, she will wonder why I have them. (Male, 68 years)

It’s embarrassing to put it on and tell your partner that ‘Today we are using a condom’. She will ask me ‘What’s going on today?’. Even if she could come one day with a female condom, and say ‘Today we’ll use the condom’, it’s a fight right there. (Male, 70 years)

One male participant responded that he had used a condom for a long time and that it should not cause a conflict:

That it causes fights, I don’t think so, because when I started to use the condom, it was 1955–1955! That time, AIDS was not there. I used to love women … even now, I still love women. (Male, 83 years)

The same concerns of faithfulness were observed with female participants:

These old men don’t want to use condoms. He will say ‘Why now?’ and accuse me of cheating … (Female, 68 years)

I wouldn’t agree to the condom … he was the unfaithful one, not me. He was the unfaithful one. We shouldn’t accept these things. (Female, 75 years)

One female was open to using a female condom, although another was particularly concerned about side-effects:

If there was a condom for women, yes, so that women could protect themselves. (Female, 60 years)

I don’t prefer the femidom, even if I wanted to fool around with someone. As women, we get sebabo [vaginal thrush], then I insert the femidom over the thrush. The partner is not wearing a condom, he just fiddles around until he goes in. You won’t even see what is going on because you are lying there, and the thing will be going over that thrush. No ladies, let’s stop all this nonsense. (Female, 62 years)

### 3.4. Sexual prowess and double standards

Male participants expressed tendencies to exert their sexual prowess, whereas some females seemed to accept that men are promiscuous:

… and you lust after her, after her figure … another one comes with a smaller figure, and you compare. It’s admiring a lot of women. … . (Male, 68 years)

I used to love women, even now I still love women … and I got [have] my taste. I never slept with a woman who’s not my choice, no! That was my choice, we are men mos … (Male, 83 years)

I can sleep with two women here, or three to four, take my car, go to Mabopane, take my car, go to Klipgat, until it becomes late. By nine, ten o’clock I’m still sleeping with them … so long as I’m strong. (Male, 65 years)

I am still with my husband. I noticed that he still looks around. What can we do? Men love women … we must just accept. If he still wants to venture out there, he’d rather go and do those things out there, I told him that I no longer have interest in sex, I don’t want him to bring me diseases … (Female, 75 years)

### 3.5. Economic status

Male participants seem to think that if they have money, they can sleep with women. They even quantify women as ‘cheap’, as one can pay a minimal amount of money to have sex with them. Older women were silent on the issue of money:

Money, If I have money, I can have lots of wives daily … if I have money, I can sleep with a lot of women … (Male, 83 years)

, You then use the money to lure her, ‘Let’s go get some Kentucky’ … You take her out … a woman is a woman. You know, our women, they are very cheap. Cheap, cheap, cheap. (Male, 87 years)

The money issue, you cannot rule it out. Right now, do you know that our sisters, girls, they have given themselves to these foreigners, and these foreigners are using them. They bribe these girls with money and when they leave them … that’s why you find that our boys, when they are involved with these girls, when the girls leave, they don’t tell. (Male, 80 years)

## 4. Discussion

The current study focuses on exploring gender perceptions of risk of HIV infection among older persons. Many of the findings are similar to studies conducted elsewhere, mostly in younger populations due to the paucity of research in the older population. Findings show that there are important differences in perceptions between men and women as to the underlying mechanism of HIV/AIDS. These stem from sexual behaviours and socially constructed gender differences between men and women in their roles. Lotfi, Ramezani, Tehrani, Merghati Khoei, Yaghmaei and Dworkin (2013) are of the opinion that risk of HIV infection can originate in women’s behaviour or her husband’s behaviour, or both.

In this study, both genders blame each other for the spread of HIV infection. Elderly women in this study blame men for spreading HIV by having a sexual encounter with younger women, while men believe that some women deliberately infect men. This position has been found in many studies. Literature has shown that gender stereotypes allow women to be blamed for spreading HIV infection (Rankin *et al*. 2005). Men are often reported to be infected by women, who may be castigated by men and women alike, while less blame tends to fall on men as opposed to women who have multiple partners (Peacock *et al*. 2008). If HIV infection is discovered in a wife first, she is readily blamed by her husband, and in some instances, women may equally be blamed by other relatives, regardless of whose infection was discovered first (Peacock *et al.* 2008). As Mbonu, Van den Borne and De Vries (2010) note, men and women who are infected with HIV may suffer from the same illness, but society’s predisposition to the assumed subservient position of women may expose them to an extremely negative response. The vulnerability paradigm also rests on the assumption that men are more likely than women to bring HIV into the partnership (Higgins, Hoffman, Shari & Dworkin 2010). The fear of being blamed for infecting partners is to an extent responsible for lack of sharing of HIV status with partners (Peacock *et al*. 2008; Söderström 2006; Tagwirei 2014). Tagwirei (2014) asserts that blaming either sex serves to reinforce stigma against a section of the community which is already suffering the scourge of the epidemic.

Mixed responses to disclosure were prominent in this study. Some male participants believed that HIV-positive people would conceal their status and deny it, while others supported disclosure. The elderly men feared that they would be gossiped about if people knew their positive status. This reluctance to disclose as a result of concern about gossiping among men was also found in a study by Deribe *et al*. (2010). Female participants were open to disclosure, particularly if it was done by their doctors in the presence of their partners. Studies among women show that women are less likely to disclose their status, to avoid social isolation (Iwelunmor, Airhihenbuwa, Okoror, Brown & Belue 2006). Disclosure has been associated with a potential risk of stigmatisation, fear of rejection, abandonment, abuse and violence (Deribe, Woldemichael, Wondafrash, Haile & Amberbir 2008; Kehler, Mthembu, Ngubane-Zungu & Mtambo 2012; Mucheto, Chadambuka, Shambira, Tsimanga, Gombe & Nyamayaro 2011; Simbayi, Kalichman, Strebel, Cloete, Henda & Mqeketo 2007).

A large body of evidence indicates that women’s economic and social vulnerability relative to men means that disclosure remains a barrier in many parts of sub-Sahara Africa. In a recent South African study, disclosure to sexual partner was found to be higher among women whilst men chose to remain silent about their HIV status (Tshweneagae, Oss & Mgutshini 2015).

Men and women in this study had diverse perceptions of the use of condoms. Both men and women were concerned about introducing the use of condoms with their elderly partners at their advanced age, as their faithfulness would be questioned. Peacock *et al*. (2008) posit that the uptake of condom usage is still problematic, as men see health-seeking behaviour as weak, and women may be seen as ‘easy’ and unfaithful if they carry condoms. As for older women, condom uptake is further unappreciated among this group as concerns about pregnancy are not an issue, and their perceptions are that their male partners control condom use (Nguyen & Holodniy 2008). Furthermore, there are difficulties in communication between partners: asking a partner to use a condom suggests that he has been unfaithful, and may also suggest that the woman has been cheating (Nguyen & Holodniy 2008; Söderström 2006). According to Scott (2009), the issue of trust is vital, since requesting the use of condoms may provoke suspicions. One elderly woman had challenges with the use of the female condom. Research shows that rejection of condom use is due to several reasons, including physical side effects (Versteeg & Murray 2008).

Elderly men were not shy to expose details of their sexual prowess, and the fact that as men they can engage in multiple sexual encounters. Studies show that constructions of masculinity also encourage men to have multiple concurrent sexual partners, while placing a high value on female fidelity (Falola & Heaton 2007; Haruna & Ago 2014; Peacock *et al*. 2008:1). In several developing countries, male sexual prowess is highly valued, and that these culturally prescribed acts of sexual promiscuity are potentially laden with risk of HIV infection (Barrett, Moya & Liddell 2005; Keetile 2014).

In South Africa, multiple partnerships are condoned and even encouraged for men, while women are expected to be monogamous and unquestioning of their partner’s behaviour (Mswela 2009). In this study, older men stated that they lust after women and that because they are men, they can have as many partners as they can, implying that they cannot help themselves. A similar study on sexual and reproductive health of South African men shows than men tend to lack self-control and feel that they are invincible, which could undermine their HIV risk perception (Stern *et al*. 2014). Older women in this study felt relegated to accepting that men have the right to multiple partners. This phenomenon was also found in a study by Lotfi *et al*. (2013), where women experienced and faced double standards and it was normative for men to have multiple sexual partners.

Findings in this study indicate how women are complicit with hegemonic masculine norms. For example, some women endorsed the idea that men can indulge in extramarital sex once they experience menopause. This is a social construction designed for women in African tradition, according to Baloyi (2009), where sexual intercourse is not expected to be a long-life practice for women, and when they enter menopause, they are no longer considered suitable to have sex. Some sociocultural factors in developing countries indeed include toleration of male promiscuity and resistance to fidelity (Barrett *et al*. 2005; Dako-Gyeke 2013). It is not therefore surprising that older men are reported to be more sexually active than older women (Nguyen & Holodniy, 2008).

Poverty is another driving force of HIV transmission in women (Joint United Nations Programme on HIV/AIDS 2012). Male participants also stated that they can sleep with women in exchange for money. They explained that women are ‘cheap’ and can be bought with anything, including food. This attests to Hellandendu’s (2012) belief that females yield due to power relations and economic disparities, because of the large material rewards they receive from men. Findings were therefore consistent with previous research that underscores how women’s limited financial autonomy may constrain them into sexually risky behaviour (Dworkin & Blankenship 2009; Dworkin, Kambou, Sutherland, Moalla & Kapoor 2009). The power imbalance between men and women also translates into economic dependency for women.

## 5. Conclusion

Culturally sanctioned gender relations play an especially prominent role in the HIV/AIDS epidemic in sub-Saharan Africa, where HIV infection rates among women substantially exceed those among men (Joint United Nations Programme on HIV/AIDS 2008). Women are frequently blamed as vectors of HIV transmission, although this is contrary to the facts. In this study, the blame for transmitting HIV infection seems to be allocated in both directions, with males and females blaming each other. This study confirms the pervasive notion of the promotion of double standards for males. Elderly men seemed to increase their clout according to the number of relationships they can have by virtue of being men, while women felt relegated to accepting that men have the right to multiple partners.

Most societies in Africa expect their women to be monogamous but expect men to have extramarital affairs (Rankin *et al.* 2005). These traditional social values condone male sexual promiscuity while placing a high value on female fidelity (Haruna & Ago 2014). These cultural practices place women at a particular disadvantage regarding safe sex (Scott 2009). Some older females promoted extramarital relationships, as they felt that they have reached menopause and have no sexual desire. This confirms the opinion in some traditional African cultures that sex has mainly been for the pleasure of the ‘man’, and that women should not have sex after menopause (Baloyi 2009; Mofolo 2010).

Male participants were opposed to introducing a condom with their wives, as they feared that their fidelity would be questioned. Females claimed that men do not want to use condoms. Evidence shows that condom use within marriage suggests lack of trust between partners and consequently betrays the intimacy that is necessary within a marital relationship. A study in Malawi considered condom use in marriage as an ‘intruder’ in the domestic space (Chimbiri 2007). The risk of HIV in marriage is directly linked to non-use of condoms (Shisana, Zungu-Dirwayi, Toefy, Simbayi, Malik & Zuma 2004). Mixed opinions about disclosure were noted in this study. Men believed that those infected would not disclose to their partners. Deribe *et al*. (2010) in their study found that men were more likely than women to report that they did not disclose to their partners because they did not want to reveal infidelity The decision not to disclose can negatively impact the prevention of HIV transmission to partners (Bott & Obermeyer 2013). In some African settings, policy-makers and women groups have supported criminalising transmission as a way to change male behaviour and punish men who transmit HIV to female partners (Joint United Nations Programme on HIV/AIDS & United Nations Development Programme 2008). Female participants were open to disclosure.

Male participants seemed to think that women can offer sex, and at a low cost, because of poverty. This perception of the men supports the fact that unequal gender relations and unequal access to economic resources mean that women tend to be poorer than men. According to Scott (2009), a number of socio-economic factors, which include the difficulty that women face in finding regular employment, have an impact on HIV transmission.

## 6. Recommendations

The recommendations below flow from the conclusions of this study:

1. There is a need develop target-specific, culturally relevant behavioural intervention programmes for substantial behaviour change for the elderly.
2. There is clearly a need for more investment in health sector initiatives to encourage voluntary testing and disclosure of HIV status. The focus of health education should be on strategies to assist the elderly to disclose their status.
3. Prevention interventions based on social and cultural realities need to be sought to empower elderly women.
4. Women should be educated at an early age that they do not need to be complicit with hegemonic masculine norms.
5. Equally, platforms need to be created for men to reflect on changing cultural practices that undermine women and perpetuate male dominance.
6. Age-appropriate advertising campaigns targeting older persons should form part of the education campaign, particularly regarding prevention issues such as condom use.
7. Ways to embrace men’s susceptibility to HIV infection while simultaneously addressing their gender privileges need to be sought.
8. More in-depth and extensive research in this area is recommended.
